# Electroless Nickel Deposition for Front Side Metallization of Silicon Solar Cells

**DOI:** 10.3390/ma10080942

**Published:** 2017-08-14

**Authors:** Shu Huei Hsieh, Jhong Min Hsieh, Wen Jauh Chen, Chia Chih Chuang

**Affiliations:** 1 Department of Materials Science and Engineering, National Formosa University, 64, Wunhua Road, Huwei, Yunlin 632, Taiwan; shhsieh@nfu.edu.tw; 2 Graduate School of Materials Science, National Yunlin University of Science and Technology, 123 University Road, Section 3, Douliou, Yunlin 640, Taiwan; m10347008@yuntech.edu.tw; 3 Motech Industries Inc., No.2, Dashun 9th Rd., Xinshi Dist., Tainan 741, Taiwan; cc_chuang@motech.com.tw

**Keywords:** solar cell, electroless, Cu/Ni contact, open circuit voltage

## Abstract

In this work, nickel thin films were deposited on texture silicon by electroless plated deposition. The electroless-deposited Ni layers were characterized by scanning electron microscopy (SEM), transmission electron microscopy (TEM), energy dispersive x-ray spectroscopy (EDS), X-ray diffraction analysis (XRD), and sheet resistance measurement. The results indicate that the dominant phase was Ni_2_Si and NiSi in samples annealed at 300–800 °C. Sheet resistance values were found to correlate well with the surface morphology obtained by SEM and the results of XRD diffraction. The Cu/Ni contact system was used to fabricate solar cells by using two different activating baths. The open circuit voltage (Voc) of the Cu/Ni samples, before and after annealing, was measured under air mass (AM) 1.5 conditions to determine solar cell properties. The results show that open circuit voltage of a solar cell can be enhanced when the activation solution incorporated hydrofluoric acid (HF). This is mainly attributed to the native silicon oxide layer that can be decreased and/or removed by HF with the corresponding reduction of series resistance.

## 1. Introduction

Sustainability has become a major keyword in the 21st century. The sustainable development of renewable energy resources to meet growing energy requirements is the most critical challenge of the modern world. Among the various renewable energy sources, solar cells are the most required due to their safe, clean, and pollution-free application. Silicon (Si) solar cells have attracted growing attention due to the reduced costs of raw materials and large-scale production. These mass-produced solar cells are usually based on crystalline Si wafers. To date, crystalline silicon solar cells have been widely used as industrial solar cells. In 1975, screen-printing was applied to solar cells for the formation of front and rear contacts [1]. Currently, the front contact and rear side of screen-printed contacts are considered to be a standard technology for metallization in solar cell manufacturing. The processes simplicity and lower processing time leads to the high-throughput production of c-Si solar cells. Conversion efficiency enhancement is a major challenge for Si solar cells. This is due to optical losses for top-contact coverage shading, higher contact resistance, and poor surface recombination that limit solar cell efficiency. Their high cost and decreasing silicon wafer thicknesses encourages silicon solar cell manufacturers to develop fresh metallization techniques that involve a lower quantity of silver usage without the need to rely on the pressing process of screen-printing. A 2017 international technology roadmap for photovoltaics (ITRPV) demands a superior performance with a lower usage of silver pastes for the future of metallization [2]. In this regard, a great deal of investigation has already been conducted on different metallization techniques as an alternative to screen-printing technology. The minor production costs and higher efficiencies of metallization techniques are critical for the progression of solar cell manufacturing technology. Among the promising metallization techniques, low-cost Cu/Ni plating techniques are emerging as a potential solution to higher metallization costs, as well as a route to establishing further improvements in cell performance [3,4,5]. These Cu/Ni plating techniques can be realized at lower material costs and are suitable for use in mass production.

The main benefits of Cu are its low cost and high conductivity. However, it has a high diffusivity and solubility in Si, and Cu contamination in solar cells can degrade cell performance by introducing efficient minority carrier recombination sites [6,7,8]. It is necessary to implement a diffusion barrier between Cu and Si to prevent inter-diffusion deterioration in devices. A thin layer of nickel (Ni), deposited on the contact areas, is typically used as a Cu diffusion barrier [9]. The standard processing sequence of Ni/Cu contact formation involves two steps: in the first step, (i) a Ni metal film is plated onto the solar cell surface, followed by (ii) the final step of plating a copper metal film on top of the Ni [5]. Evaporation, sputtering, and electrochemical deposition techniques have been applied for integrated circuit (IC) manufacturing. Electroplating of metals is well established for the metallization of semiconductor devices, and presents several advantageous properties that make it of interest for the front side metallization of silicon solar cells. Electroplating of metals involves the transfer of electrons between electrodes and an electrolyte containing the metal ions. Electroplating offers the highest degree of control and the fastest plating rates since the source of electrons is directly controlled by an external power supply. However, the formation of metal contacts using electroplating processes can only be used for conductors. Therefore, electroplating requires a seed layer with a certain conductivity to minimize the potential drop with increasing distance from the contact point(s) and to achieve uniform deposits. On the other hand, electroless plating enables the direct plating of metal on Si. Electroless plating technique has been applied for ohmic contacts to n- and p-doped silicon [10]. Mandelkorn et al. combined photolithography patterning and electroless Ni plating to define the front side contacts of silicon solar cells [11]. Electroless nickel deposition is an autocatalytic process achieved by the immersion of a substrate in a plating bath. It is based on the capture of electrons by the cations present in the solution, which are absorbed on the surface of the substrate. These electrons are supplied by a reducing agent dissolved in the solution. In our case, the main electron source is the oxidation of the sodium hypophosphite (NaH_2_PO_2_). The nickel ions are reduced to solid nickel on top of the silicon. The deposit is not pure nickel, but contains phosphorous [12]. Prior to the electroless plating process, the pretreatment of the silicon surface is required. The pretreatment of Si consists of sensitization in a solution of SnCl_2_ and HCl, and activation by the deposition of Pd in a solution of PdCl_2_ and HCl. Finally, the electroless Ni seed layer is thickened by Ni and Cu electroplating. Little work has been conducted on the interfacial reactions of electroless nickel thin films on textured silicon. In the present paper, a front-side metallization was produced by Ni-P electroless deposition and Ni/Cu electroplating on crystalline silicon solar cells. The interfacial reactions of electroless nickel thin films on textured silicon were investigated. Two activating baths were used for evaluations of the open circuit voltage (Voc) of the solar cell. The solar cells were fabricated with two different activating baths (with and without HF). These solar cells were annealed isothermally in a furnace at 500 °C for different periods in an Ar/H_2_ atmosphere to study the effects of pre-treatment on the open circuit voltage (Voc) of the solar cell.

## 2. Results and Discussion

**Figure 1:**
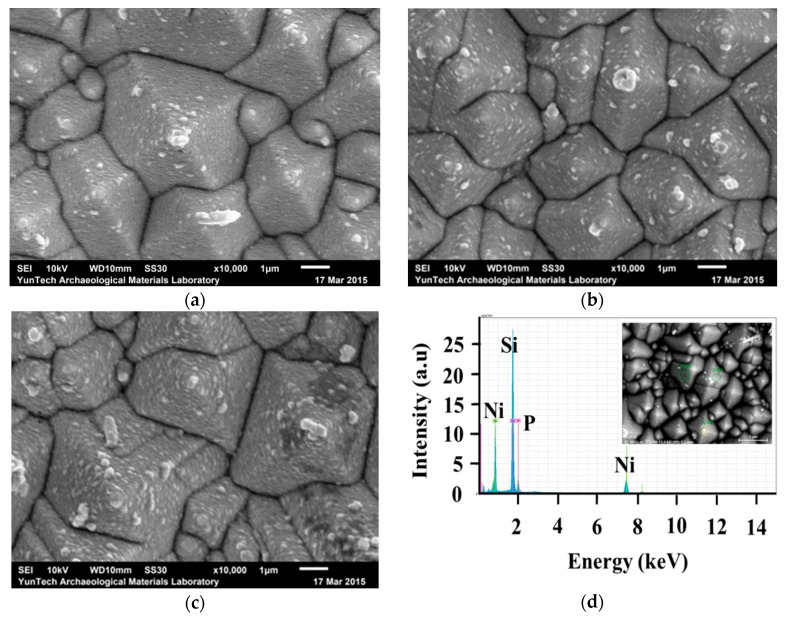

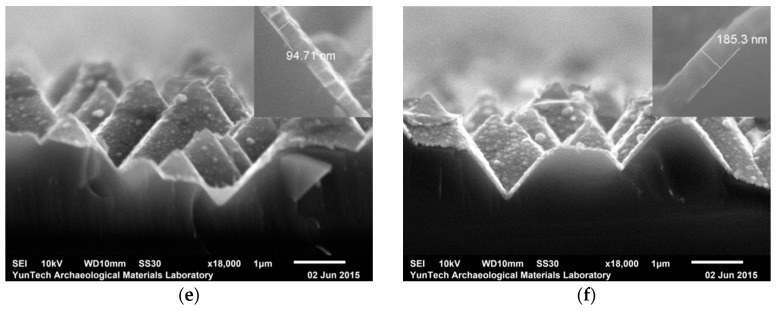
SEM images of samples with deposited electroless Ni at 70 °C for (**a**) 60; (**b**) 75; and (**c**) 120 s, respectively; (**d**) EDS diagram for the Ni deposit plated for 60 s; SEM images (cross-section) of samples with deposited electroless Ni at 70 °C for (**e**) 90 s and (**f**) 180 s, respectively; The insets in (**e**) and (**f**) show the thickness of the electroless Ni.

Some of the most important issues for metal deposition are adhesion and surface coverage. Figure 1a–c show the SEM images of samples with deposited electroless Ni at 70 °C for 60, 75, and 120 s, respectively. The coating surface is smooth due to the formation of many tiny particles during electroless deposition. A uniform and texturized surface coverage is achieved and the deposits are homogeneous. As can be seen in the SEM images in Figure 1, for a short plating time (60 s), all the pyramids are metallized. The EDS diagram for the electroless Ni deposit plated for 60 s with a pH of 5 at 70 °C is shown in Figure 1d. The elemental SEM/EDS analysis shows the presence of silicon, nickel, and phosphorus, thus suggesting the success of electroless nickel plating. The peak for Si is of course from the Si substrate. Figure 1e,f show the SEM cross-section views of samples with deposited electroless Ni at 70 °C for 90 and 180 s, respectively. The insets in (e) and (f) show the thickness of electroless Ni. The thickness of electroless Ni deposits with a plating time of 60 s, 90 s, 120 s, and 180 s are 58 nm, 95 nm, 106 nm, and 184 nm, respectively. The electroless Ni deposition rate is about 1 nm/s. The deposits are continuous thin films above the thickness of 45 nm. The characteristics of this bath enable us to conclude that it can be used as a seed layer and diffusion barrier before copper deposition for contact solar cells on silicon. 

**Figure 2:**
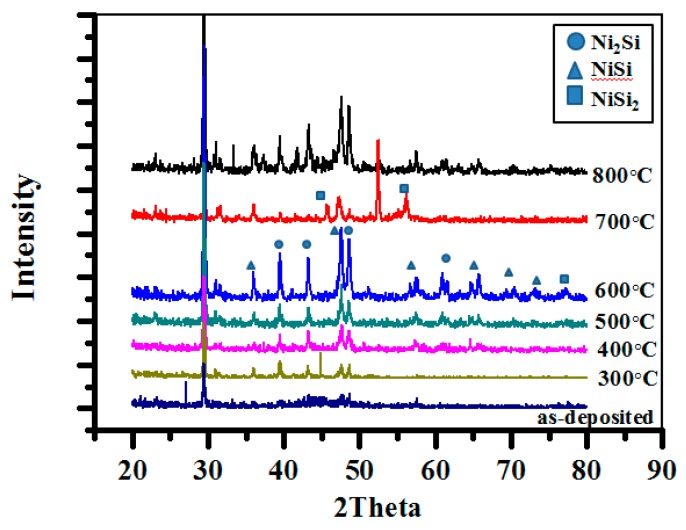
XRD patterns of electroless Ni deposits plated for 60 s with a pH of 5 at 70 °C: samples without annealing and those annealed at 300–800 °C for 10 min.

To obtain more information about the phase formation during the annealing, XRD analyses were carried out. Figure 2 shows the XRD patterns of electroless Ni deposits that were plated for 60 s with a pH of 5 at 70 °C both without annealing and annealed at 300–800 °C for 10 min. The XRD analysis of the samples after annealing at 300–600 °C shows almost exactly the same pattern except for some changes in relative intensities. By comparing the XRD peaks with the cards of Joint Committee on Powder Diffraction standards (JCPDS), the peaks at (2θ) 30.87, 39.48, 43.29, and 48.52° correspond to Ni_2_Si (210), Ni_2_Si (211), Ni_2_Si (021), and Ni_2_Si (002), respectively. Additionally, the peaks at (2θ) 31.51, 35.95, and 47.53° correspond to NiSi (002), NiSi (111), and NiSi (211), respectively. Based on these XRD spectra, Ni_2_Si and NiSi are present up to 800 °C. The XRD pattern of the sample annealed at 700 °C revealed NiSi_2_ peaks in addition to the peaks corresponding to Ni_2_Si and NiSi phases. This indicated that Ni_2_Si, NiSi, and NiSi_2_ exist at temperatures higher than 700 °C. A strong peak around 54 degrees corresponds to Si (311). The lack of peaks above 60 degrees is due to the strong peak of Si (311). The high intensity of the Si (311) peak covers the peaks above 60 degrees. Also, the d-spacing of Ni (111) is the same as that of Ni_2_Si (021). Therefore, it is hard to find the formation of the possible recrystallization of nanocrystalline Ni(P) films from the XRD spectrum. The formation of Ni_3_P phases are possible in electroless nickel thin film after annealing. However, no diffraction peaks from Ni_3_P is observed within the detection limit.

In general, the nickel thin films on the surface polished silicon via the vacuum plating process are heated and nickel silicides are formed; the phase of the nickel silicide depends on the temperature of formation. Ni_2_Si forms at approximately 200 °C. Upon further heat treatment, the monosilicide, NiSi, forms at about 300 °C and the disilicide, NiSi_2_, at about 700 °C [13]. There is evidence that the Ni atoms are the major moving species during the growth of Ni_2_Si, NiSi, and NiSi_2_ [14]. As mentioned above, Ni_2_Si and NiSi were detected in samples annealing at 300–800 °C. This indicated that the silicide formation sequence is different from the polished silicon case. The Ni_2_Si phase that still remained in samples annealing at 300–800 °C may be attributed to the oxide interlayer between the electroless nickel thin films and the texture silicon. Mondon et al. [15] reported that, despite the HF-dip prior to nickel plating, an interfacial oxide layer was found between nickel and silicon. Native oxide seems to regrow quickly up to a thickness that effectively hinders silicide formation. Lee et al. [16] suggested that as native oxide serves as an effective kinetic diffusion barrier for the reaction between Ni and Si substrates, it retards the supply of Ni and affects the silicides formation kinetics. This is considered the reason for two silicide phases coexisting at an annealing temperature of 300–800 °C. 

**Figure 3:**
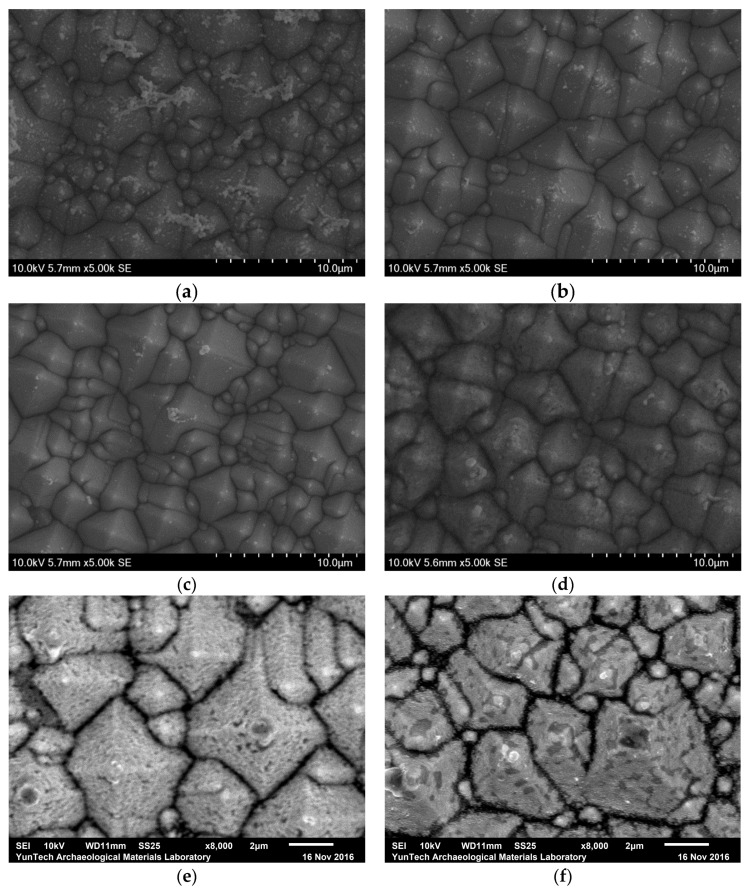
SEM images of electroless Ni/Si films plated for 60 s, including samples after thermal annealing at (**a**) 300 °C; (**b**) 400 °C; (**c**) 500 °C; (**d**) 600 °C; (**e**) 700 °C; and (**f**) 800 °C for 10 min.

The SEM images of electroless Ni/Si films plated for 60 s, including samples after thermal annealing at 300–800 °C for 10 min, are shown in Figure 3. After annealing at 300–600 °C, the surface morphology of electroless Ni/Si films does not change and the smooth surface morphology appears to be preserved. This showed that Ni_2_Si and NiSi are very stable up to 600 °C. After annealing at 700 °C for 10 min, a small amount of gray area is seen on the surface. The gray area was found to increase for the sample annealing at 800°C, reflecting the more unstable nickel silicide layer in this sample.

**Figure 4:**
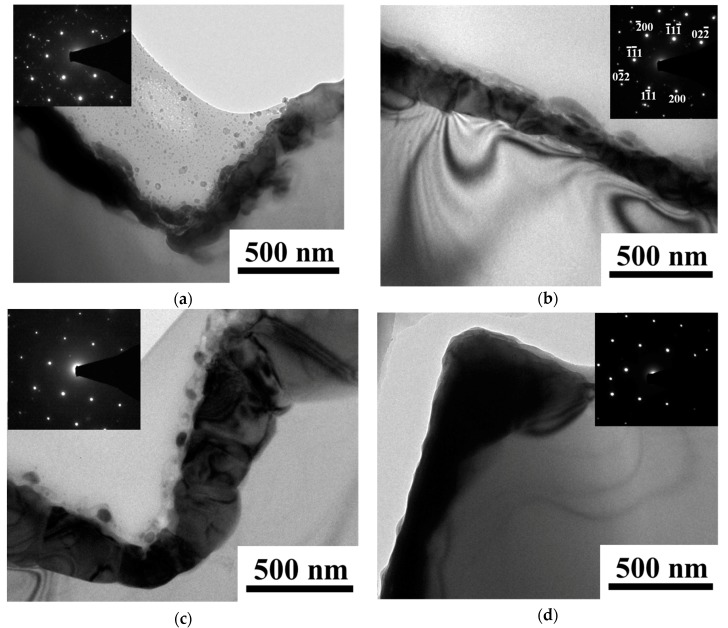
TEM images of electroless Ni/Si films plated for 60 s, including samples after thermal annealing at (**a**) 500 °C; (**b**) 600 °C; (**c**) 700 °C; and (**d**) 800 °C for 10 min.

Figure 4 shows TEM images of electroless Ni/Si films plated for 60 s, including samples after thermal annealing at 500–800 °C for 10 min. The insets display the corresponding selected area electron diffraction (SAED) pattern for the nickel silicides film and silicon substrate. These figures show a set of sharp and regular spots that clearly reveal the single crystalline and cubic phase of the silicon. The (200) reflection is a double diffraction. The other spots reveal the phase of nickel silicides. Polycrystalline Ni_2_Si and NiSi were found to be present in samples annealed at 500 and 600 °C. The thickness of the silicide layer is about 125–160 nm. For the sample annealed at 700 °C, the thickness of the silicide layer increases to 210–290 nm in some areas. In general, silicides are formed usually by means of reactions between a deposited metal layer and the silicon substrate. Nickel is the dominant diffusing species during the formation of nickel silicides. For the nickel layer with a thickness of 60 nm, the thickness of NiSi and NiSi_2_ are about 120 nm and 180 nm, respectively. This reveals the formation of NiSi_2_ and of unstable nickel silicides after annealing at 700 °C. Agglomeration of NiSi_2_ is observed for the sample after annealing at 800 °C. This suggests that the films became unstable at temperatures higher than 700 °C. These results are in agreement with the results of SEM.

**Figure 5:**
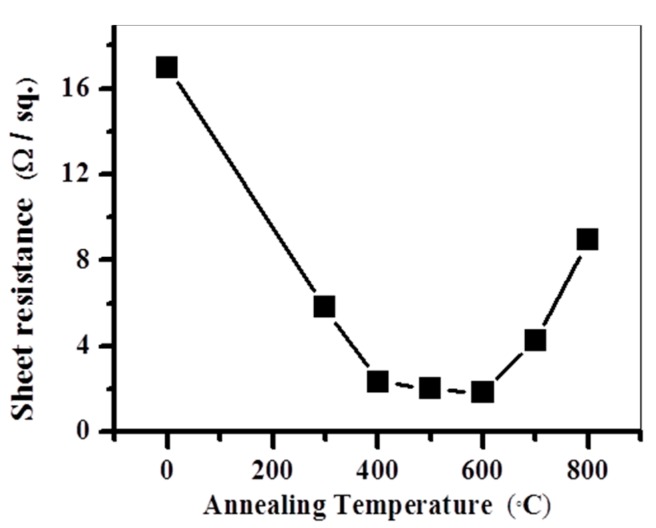
Sheet resistance of electroless Ni (60 nm)/Si films as a function of annealing temperature.

Figure 5 shows the sheet resistance of electroless Ni (60 nm)/Si films as a function of the annealing temperature. The phase formation can be also assessed by sheet resistance measurements. The average value of the sheet resistance came down after annealing compared to the as-deposited sample. The sheet resistances have a downward trend with an increase in annealing temperatures up to 600 °C. This downward trend in sheet resistance can be ascribed to the formation of Ni_2_Si and NiSi. However, an abrupt increase was observed after annealing at 700 °C. The results of sheet resistances and SEM surface views of the samples annealed at various temperatures and the abrupt increase of sheet resistance near 700 °C can be ascribed to the unstable quality of the silicides layers of the samples. 

**Figure 6:**
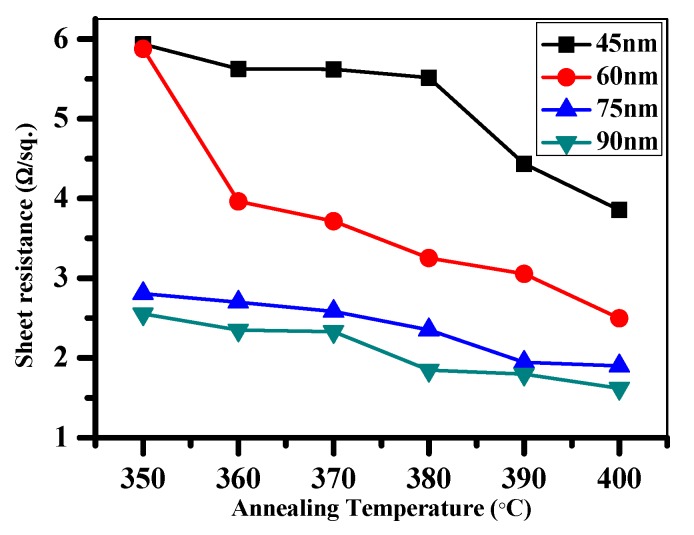
Sheet resistance as a function of annealing temperature at 350–400 °C for the Ni (45 nm)/Si, Ni (60 nm)/Si, Ni (75 nm)/Si, and Ni (90 nm)/Si samples.

To obtain deeper insight into the information affecting photovoltaic performance, heat treatment for various thicknesses of electroless Ni films were performed. Figure 6 plots the sheet resistance as a function of annealing temperature between 350–400 °C for the Ni (45 nm)/Si, Ni (60 nm)/Si, Ni (75 nm)/Si, and Ni (90 nm)/Si samples. The sheet resistances of all the samples decrease slightly as the annealing temperature increases. The decrease in sheet resistance may be attributed to the greater NiSi phase that occurs during thermal annealing. 

**Figure 7:**
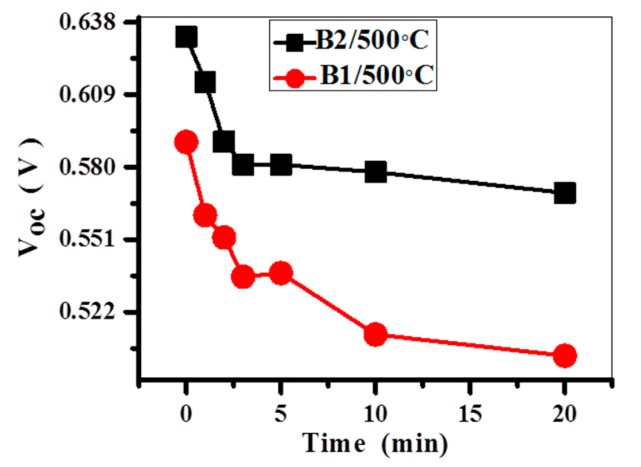
Open circuit voltage as a function of the annealing times for the samples prepared by using bath 1 (B1) and bath 2 (B2).

As mentioned above, the formation of an oxide layer on the wafer surface is inevitable during sensitization and activation. To understand the effect of the oxide layer on the Voc of a solar cell, two different activating baths were used in this study. The open circuit voltage Voc of the Cu/Ni/Si samples with two different activation treatments of finger pattern silicon, before and after annealing, was measured under AM 1.5 conditions to determine the solar cell properties. Bath 1 is the conventional activating bath, PdCl_2_/HCl (0.15 g/L PdCl_2_ + 3 mL/L HCl), as reported in the literature [14], which is denoted B1. Bath 2 is the activation solution with HF (0.15 g/L PdCl_2_ + 3 mL/L HCl + 83 mL/L HF), which is denoted B2. Figure 7 shows Voc as a function of the different annealing times for samples prepared by using bath 1 and bath 2. For the solar cell prepared by using bath 1, an open circuit voltage (Voc) of 0.590 V is obtained before annealing. When the HF is incorporated, the Voc is significantly improved to 0.632 V. The open circuit voltage of the samples after annealing have a downward trend with an increase in annealing times up to 20 minutes. For the samples prepared by using bath 2, the open circuit voltage drops to 0.614, 0.590, 0.5810, 0.581, 0.578, and 0.570 V after annealing at 500 °C for 1 min, 2 min, 3 min, 5 min, 10 min, and 20 min, respectively. The Voc decreases to 97.2% and 90.2% of the original value after annealing at 500 °C for 1 min and 20 min, respectively. For the samples prepared by using bath 1, the open circuit voltage drops to 0.561, 0.552, 0.536, 0.538, 0.513, and 0.505 V after annealing at 500 °C for 1 min, 2 min, 3 min, 5 min, 10 min, and 20 min, respectively. The Voc decreases to 95.1% and 85.59% of the original value after annealing at 500 °C for 1 min and 20 min, respectively. The results show that the sample prepared by using bath 2 is more resistant to the thermal budget than the sample prepared by using bath 1. The observed improvement due to the addition of HF to the activation solution appears obvious. This is mainly attributed to the native silicon oxide layer that can be decreased and/or removed by HF with the corresponding reduction of series resistance. 

**Figure 8:**
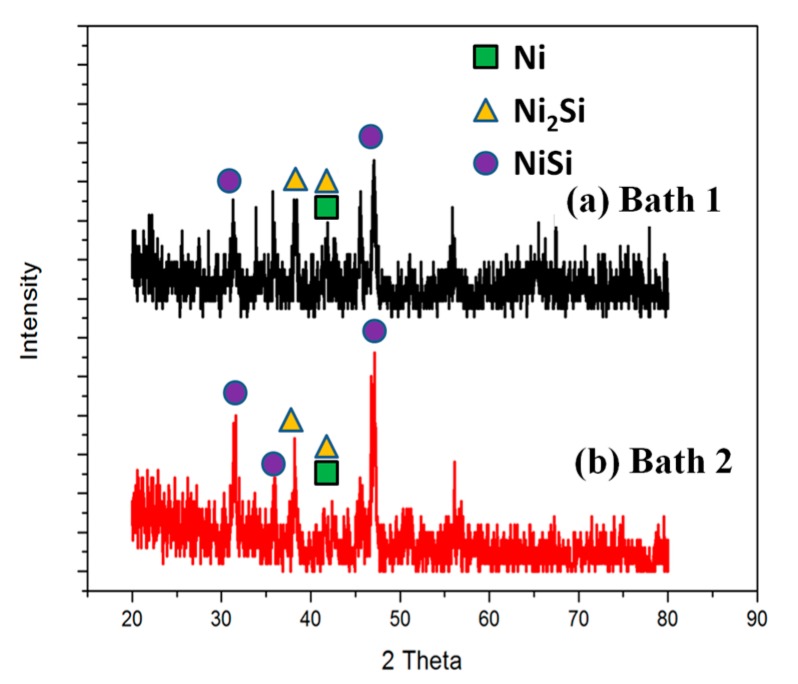
XRD patterns of the Ni/Si samples with two different activation treatments ((**a**) bath 1 and (**b**) bath 2) annealed at 400 °C for 10 min.

Figure 8 shows the XRD patterns of the Ni/Si samples with two different activation treatments (bath 1 and bath 2) annealed at 400 °C for 10 min. XRD analysis of the two samples after annealing at 400 °C shows almost the same pattern, except for some changes in relative intensities. The intensities of the NiSi peaks in Figure 8b is slightly stronger than that of the peaks in Figure 8a. This suggested that, as native oxide serves as an effective kinetic diffusion barrier for the reaction between Ni and Si substrates, it retards the supply of Ni and affects the silicides formation kinetics [16].

**Figure 9:**
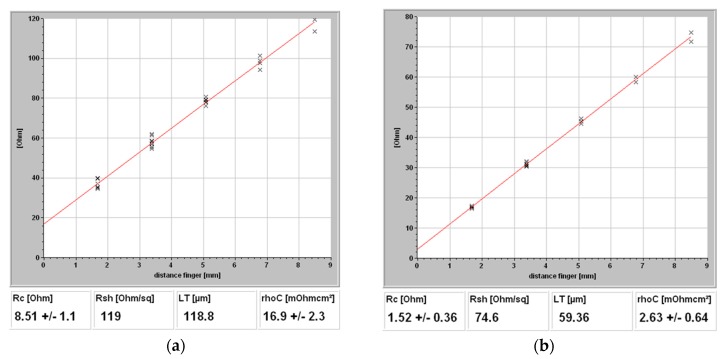
Contact resistivity of plated Ni/Cu contacts prepared by (**a**) bath 1 and (**b**) bath 2.

The contact resistivity of plated Ni/Cu contacts prepared by bath 1 and bath 2 were measured and are shown in Figure 9. For the solar cell prepared by bath 1, a contact resistivity of 17 mΩcm^2^ is obtained. When HF is incorporated (bath 2), the contact resistivity is improved to 3 mΩcm^2^. The contact resistivity of the samples prepared by bath 1 is higher than that of the samples prepared by bath 2, which indicates that the samples obtained by using bath 2 have better quality. The results suggest that the native silicon oxide layer can be decreased and/or removed by the addition of HF to the activation solution. Thus, in this study, we observed an enhanced open circuit voltage when the activation solution incorporated HF. In this work the influence of the Cu/Ni contact system on the properties of the solar cells was compared by using two different activating baths.

## 3. Materials and Methods

Two different kinds of samples were investigated in this work: test samples for the evaluation of the sheet resistance, and solar cells with Ni/Cu plated front-side metallization for open circuit voltage (Voc) measurements. Single crystal phosphorus-doped (0 0 1) oriented silicon wafers with textured roughness around 3–5 μm were used in the evaluation of the sheet resistance and structure characterization. The process of electroless Ni plating on a textured silicon surface was performed using a chemical bath, which consisted of a Ni source in the form of metal salt, NiSO_4_·6H_2_O (nickel sulfate), and NaH_2_PO_2_·H_2_O (sodium hypophosphite). Prior to the electroless plating process, it was necessary to pretreat the silicon surface. The pretreatment of Si consisted of the following steps; (1) degreasing the surface using acetone by ultrasonication; (2) cleaning in H_2_SO_4_/H_2_O_2_ solution and etching in 10 vol% HF solution; (3) sensitizing in a solution of SnCl_2_ and HCl; and (4) activating by the deposition of Pd in a solution of PdCl_2_ and HCl. The NaH_2_PO_2_ helps Ni metal educe to Ni ions in the bath. This Ni metal begins to deposit on the Si surface and further nucleates, which results in the growth of the Ni layer. The electroless Ni film was deposited on the textured Si substrate in an electroless plating bath that operates at 70 °C with pH value of 5 for a plating time of 45–180 s. Some of the as-deposited samples were annealed isothermally in a furnace at 300–800 °C in Ar/H_2_ atmosphere, and some of the as-deposited samples were annealed at 350–400 °C. Unless otherwise specified, the annealing time at each temperature was 10 min. The sheet resistance measurements were performed using a standard equispace four-point probe with a probe spacing of 40 mils. Constant currents of 0.1–1 mA were injected through two outer probes. Typical read-out voltages were measured to drop on the order of mV across the two inner probes. The contact resistivity was measured by using the transfer length method (TLM). The TLM pattern defined finger widths of about 30 μm and contact length of 10 mm. A dicing saw was used to isolate the emitter between each TLM pattern. The contact resistivity was calculated by the transfer length method using four-point probe IV-measurements at various contact combinations with varying contact distances. A JEOL scanning electron microscope (SEM) (JEOL, Tokyo, Japan) operating at 20 kV was used to collect morphological information of the samples. The elemental analysis of the samples was identified by an SEM equipped with an Oxford Link energy dispersive X-ray spectrum (EDS) (Oxford, Belfast, UK). For each EDS analysis, three points in each sample were examined and the results were averaged. A JEOL-JEM 2010 transmission electron microscope (TEM) (JEOL, Tokyo, Japan) operating at 200 kV was used for TEM examination. Powder X-ray diffraction (XRD) analyses were performed on a Bruker D8 Advance diffractometer (Bruker, Berlin, Germany) with Cu Kα radiation.

For Voc measurements of solar cell samples, silver was used for the back metal contact and Cu/Ni plating was used for the front metallization. The Cu/Ni plating process consisted of the following steps: (1) pretreatment of finger pattern silicon; (2) thin electroless nickel films, about 60 nm in thickness, were deposited onto the finger pattern silicon; (3) annealing isothermally in a furnace at 400 °C in Ar/H_2_ atmosphere for 10 minutes; (4) removing unreactive Ni films by HNO_3_; and (5) electroplating the Ni and Cu metals. The fabricated solar cells were annealed isothermally in a furnace at 500 °C in Ar/H_2_ atmosphere for 1–20 min. The fabricated device was analyzed using current–voltage (I–V) measurement under solar simulator illumination. For the investigation of the effect of the activation process on the open circuit voltage, two different activating baths were used in this study. Bath 1 was the conventional activating bath, PdCl_2_/HCl (0.15 g/L PdCl_2_ + 3 mL/L HCl), as reported in the literature [9], which is denoted B1. Bath 2 was the activation solution with HF (0.15 g/L PdCl_2_ + 3 mL/L HCl + 83 mL/L HF), which is denoted B2. 

## 4. Conclusions

In the present study, nickel thin films were deposited on texture silicon by electroless plating deposition. A drastic difference in silicide formation was found between electroless plated nickel thin films on texture silicon and vacuum plated nickel thin films on polished silicon. The dominant phases were Ni_2_Si and NiSi in samples annealed at 300–600 °C. Sheet resistance values were found to correlate well with the surface morphology obtained by SEM and the results of XRD diffraction.

The open circuit voltage Voc of Ni/Cu front metallization was affected by activation treatment. The open circuit voltage of solar cells can be enhanced when the activation solution incorporated HF. This is mainly attributed to the native silicon oxide layer, that can be decreased and/or removed by HF with the corresponding reduction of series resistance. In this work, the influence of the Cu/Ni contact system on the properties of solar cells was compared by using two different activating baths.
